# Progress and Perspective of Ceramic/Polymer Composite Solid Electrolytes for Lithium Batteries

**DOI:** 10.1002/advs.201903088

**Published:** 2020-01-21

**Authors:** Song Li, Shi‐Qi Zhang, Lu Shen, Qi Liu, Jia‐Bin Ma, Wei Lv, Yan‐Bing He, Quan‐Hong Yang

**Affiliations:** ^1^ Shenzhen Geim Graphene Center Tsinghua Shenzhen International Graduate School Tsinghua University Shenzhen 518055 P. R. China; ^2^ Laboratory of Advanced Materials School of Materials Science and Engineering Tsinghua University Beijing 100084 P. R. China; ^3^ Nanoyang Group School of Chemical Engineering and Technology Tianjin University Tianjin 300072 P. R. China

**Keywords:** interfaces, ionic conductivity, lithium batteries, solid composite electrolytes

## Abstract

Solid composite electrolytes (SCEs) that combine the advantages of solid polymer electrolytes (SPEs) and inorganic ceramic electrolytes (ICEs) present acceptable ionic conductivity, high mechanical strength, and favorable interfacial contact with electrodes, which greatly improve the electrochemical performance of all‐solid‐state batteries compared to single SPEs and ICEs. However, there are many challenges to overcome before the practical application of SCEs, including the low ionic conductivity less than 10^−3^ S cm^−1^ at ambient temperature, poor interfacial stability, and high interfacial resistance, which greatly restrict the room temperature performance. Herein, the advances of SCEs applied in all‐solid‐state lithium batteries are presented, including the Li ion migration mechanism of SCEs, the strategies to enhance the ionic conductivity of SCEs by various morphologies of ICEs, and construction methods of the low resistance and stable interfaces of SCEs with both cathode and anode. Finally, some typical applications of SCEs in lithium batteries are summarized and future development directions are prospected. This work presents how it is quite significant to further enhance the ionic conductivity of SCEs by developing the novel SPEs with the special morphology of ICEs for advanced all‐solid‐state lithium batteries.

## Graphical abstract

Herein, the advantages and ionic transport mechanisms of solid composite electrolyte (SCE) as well as the relationship between morphology of ceramic fillers and ionic conductivity of SCE are reviewed. Recent progress and strategies to settle interfacial issues for high‐performance all‐solid‐state lithium metal batteries with SCE are also concluded and future research directions of SCEs are proposed.

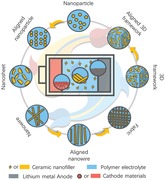

## 1 Introduction

**Figure 1:**
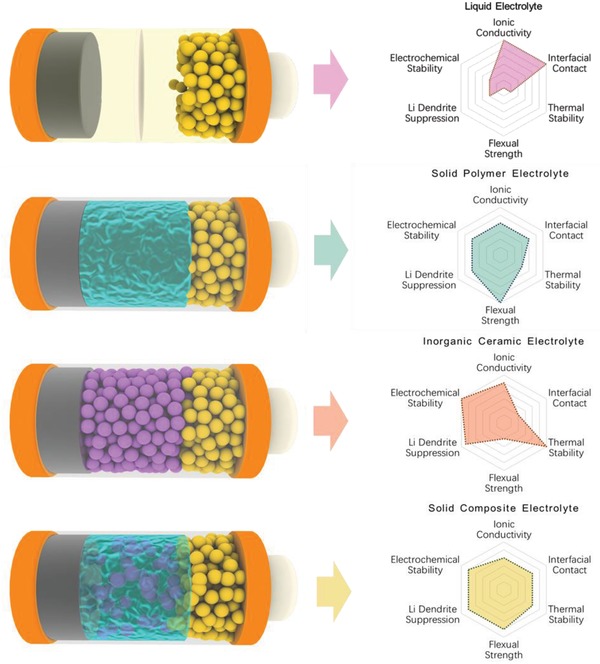
The performance comparisons of liquid electrolytes, SPEs, ICEs, and SCEs.

Energy storage devices are the most significant parts for the practical application of the renewable energy and electric vehicles (EVs), which promote the replacement of conventional power resources by renewable energy.^1^, ^2^, ^3^ As the most promising energy storage systems, lithium batteries show several advantages, such as high operating voltage, high energy density, low self‐discharge rate, and long cycle life.^4^ Therefore, they have been widely used in electronic devices, energy storage systems, and EVs. At present, the energy density of commercial lithium ion batteries with liquid electrolyte has reached ≈260 Wh kg^−1^, which is close to their limitation.^5^ Moreover, the safety concern is another huge challenge for their wide application in the EVs. The liquid electrolytes contain combustible organic solvents and thus might cause leakage and fire risks during overcharge or abused operations, especially in large‐scale applications. Therefore, replacing the liquid electrolyte with all‐solid‐state electrolyte for lithium batteries is quite necessary.^5^, ^6^ Generally, the all‐solid‐state electrolytes could be classified into solid polymer electrolytes (SPEs), inorganic ceramic electrolytes (ICEs), and solid composite electrolytes (SCEs). The SPEs consist of polymer matrix mixed with lithium salts, which consequently possess excellent process ability, flexibility, safety performance, and good interfacial contact with electrodes, but present low ionic conductivity (<10^−4^ S cm^−1^), inferior thermal and electrochemical stability, and unsatisfactory behavior in suppression of lithium dendrite growth.^7^, ^8^, ^9^ On the contrary, the ICEs show higher ionic conductivity (10^−3^–10^−2^ S cm^−1^), broad electrochemical window, and high mechanical strength, but present poor interfacial contact with electrodes.^10^, ^11^, ^12^, ^13^ Above disadvantages of SPEs and ICEs severely restrict their commercial application in power lithium batteries. The SCEs constructed by SPEs and inorganic fillers inherit the advantages of both SPEs and the inorganic fillers such as high ionic conductivity (>10^−4^ S cm^−1^), good flexibility, and intimate contact with electrodes as shown in **Figure** **1**. Therefore, the SCEs are considered one of the most promising candidate electrolytes for all‐solid‐state lithium batteries in the future.^14^, ^15^

The SCEs are composed of flexible polymer hosts, dissolved lithium salt, and rigid inorganic fillers.^3^, ^16^ Polyethylene oxide (PEO) as a typical ion‐conducting polymer host is the most dominant one compared to others such as polyvinylidene fluoride (PVDF), poly(vinylidene fluoride‐co‐hexafluoropropylene) (PVDF‐HFP), polyacrylonitrile (PAN), poly(propylene carbonate) (PPC), poly(vinyl carbonate) (PVC), and polymethyl methacrylate (PMMA).^9^, ^17^, ^18^, ^19^, ^20^, ^136^ The lithium salts such as LiN(CF_3_SO_2_)_2_ (LiTFSI),^21^, ^22^ LiN(SO_2_F)_2_ (LiFSI),^23^ and LiClO_4_ 24 are mostly applied in SPEs for their high electrochemical and thermal stability, good solvability, and promotion to formation of a stable SEI.^18^ The inorganic fillers could be classified into active fillers and passive fillers according to their ion‐conducting behavior.^9^, ^25^ The passive fillers generally include oxides (SiO_2_,^26^, ^27^ Al_2_O_3_,^28^ ZrO_2_,^29^ etc.), minerals (such as montmorillonite^30^ and halloysite^31^), carbon materials,^32^ metal organic frameworks (MOFs),^33^ etc. The active fillers commonly cover all the Li ion‐conducting materials, such as LISICON‐type (Li_14_Zn(GeO_4_)_4_), NASICON‐type (such as Li_1.5_Al_0.5_Ge_1.5_(PO_4_)_3_ [LAGP] and Li_1.3_Al_0.3_Ti_1.7_(PO_4_)_3_ [LATP]), peroskite‐type (Li_3_La_2/3−_ *_x_*TiO_3_ [LLTO]), garnet‐type (Li_7_La_3_Zr_2_O_12_ [LLZO], La‐site: Ca, Sr, Ba, K, etc., and Zr‐site: Ta, Nb, etc.), sulfide electrolyte (such as Li_10_GeP_2_S_12_), and some other ceramics (LiPON, Li_3_N, etc.).^12^, ^34^, ^35^, ^36^ Both the inactive and active fillers in polymer matrix can act as plasticizers to disorder the crystallization of polymer matrixes and thus increase the ionic conductivity of composite electrolytes, and facilitate the dissociation of lithium salts.^17^, ^25^ Moreover, the active fillers could provide highly efficient pathway for lithium ion transportation. Hence, the reinforced SCEs attract increasing attentions for their higher ionic conductivity and wider electrochemical windows. There are many reviews focused on the properties and mechanisms of passive filler‐assisted composite electrolytes.^9^, ^25^, ^37^ Regrettably, up to now, there are few articles to systematically summarize the prospects and applications of the active filler‐reinforced SCEs. In this review, we present the advances of active filler‐reinforced SCEs applied in all‐solid‐state lithium batteries and analyze the existing challenges to be conquered. The main objective of this review is to provide possible strategies to solve the problems in all‐solid‐state lithium batteries with active filler‐reinforced SCEs and highlight their inspiration for future research directions.

## 2 Ionic Conductivity of SCEs

### 2.1 Li Ion Migration Mechanism of SCEs

**Figure 2:**
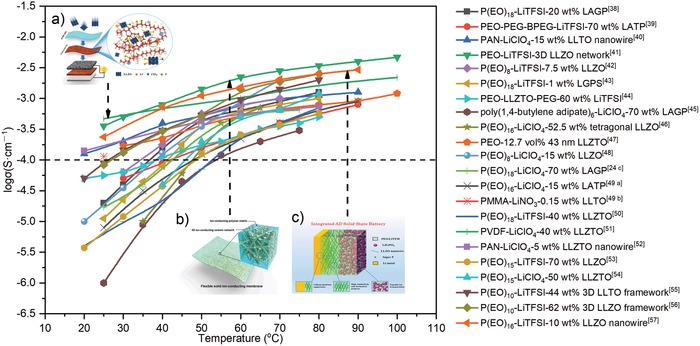
The contrast of ionic conductivity of SCEs from different refs. [qv: 24c,38–57]. a) Reproduced with permission.^41^ Copyright 2017, PNAS. b) Reproduced with permission.^51^ Copyright 2017, American Chemical Society. c) Reproduced with permission.^54^ Copyright 2018, John Wiley and Sons.

The eligible solid electrolyte applied in practical lithium batteries should possess ionic conductivity not lower than 10^−3^ S cm^−1^ at room temperature. The commonly used ICEs such as LLTO, LLZO, LAGP, and LATP could meet this requirement,^35^ while the ionic conductivity of the most SPEs is far from 10^−3^ S cm^−1^ under room temperature.^9^, ^18^ The ionic conductivity is mainly determined by the transfer mechanism. In the phase of bulk ICEs, the Li ion transfer mainly depends on the motion of vacancies or interstitial ions that results in fast ion conduction,^10^, ^35^ while the migration of ions in polymer matrix under an electric field is related to the breaks/formations of coordination bonds during the local segmental motions of polymer chains, which mainly occurs in the amorphous sections.^7^, ^18^ For the active filler‐reinforced SCEs, both of the aforementioned ion‐conducting mechanisms exist and extraordinary synergistic effects are achieved. The ceramic phase acts as plasticizer, which reduces the polymer crystallinity and increases the portion of amorphous structure and hence improves the mobility of Li ions.^27^ In addition, the acidic groups on the surface of ceramics have strong affinity with anions, which helps the dissociation of lithium salts, resulting in increased concentration of free Li ion.^31^, ^40^, ^58^ Furthermore, numerous vacancies generally exist on the surfaces of ceramics, which allows Li ions to hop among vacancies and thus provides a faster pathway than polymer electrolytes.^40^, ^52^, ^59^, ^60^ At last, the high ionic conductivity of ICEs also could effectively enhance the ionic conductivity of SCEs.^55^ However, as the intrinsic low ionic conductivity of SPEs limits the large enhancement of the total ionic conductivity of SCEs. As a result, the ionic conductivity of SCEs is still not sufficient for practical application in all‐solid‐state battery at room temperature. **Figure** **2** summarizes the ionic conductivity of SCEs reported in previous researches. It could be seen that the ionic conductivity of most as‐prepared samples is lower than 10^−4^ S cm^−1^ in the temperature ranging from 20 to 40 °C, while that of some samples could reach the order of 10^−4^ S cm^−1^. The conductivity evolution demonstrates that the strategies to take advantages of both SPEs and ICEs may be the best way to construct the SCEs for all‐solid‐state batteries with higher performance at wide working temperature.

**Figure 3:**
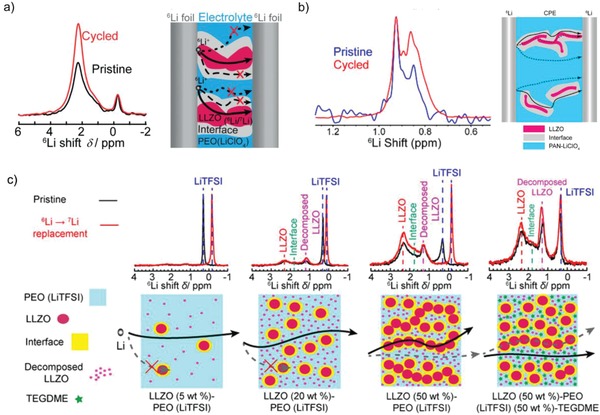
Possible ion‐conduction pathways and evidences for different composite electrolyte systems: a) LLZO‐P(EO)_18_/LiClO_4_ electrolyte. Reproduced with permission.^62^ Copyright 2016, John Wiley and Sons. b) LLZO nanowire‐PAN/LiClO_4_ electrolyte. Reproduced with permission.^52^ Copyright 2017, American Chemical Society. c) 5, 20, and 50 wt% LLZO‐P(EO)_18_/LiTFSI and 50 wt% LLZO‐P(EO)_18_/LiTFSI with TEGDME. Reproduced with permission.^64^ Copyright 2018, American Chemical Society.

Tracing the motion paths of Li ions in SCEs gives an insight into enhancement of the ion‐transfer mechanism, which is beneficial to the structure design of SCEs.^61^ The pathways for Li ion transport in SCEs involve the polymer phase, ceramic bulk phase, and interface region between SPEs and ICEs. It is still not clear to determine the dominated pathway during Li ion migration. Great efforts have been devoted to revealing the truth about Li ion migration.^52^, ^53^, ^62^, ^63^, ^64^ Hu and co‐workers^62^ tracked the transport pathways of lithium ions in 50 wt% LLZO‐P(EO)_18_/LiClO_4_ electrolyte using selective isotope labeling and high‐resolution solid‐state Li NMR. They found that upon cycling of the symmetric battery (^6^Li/LLZO‐P(EO)_18_/LiClO_4_/^6^Li), the replacement of ^7^Li by ^6^Li ions mainly occurred in the LLZO ceramic phase and the Li ion migration of SCEs is governed by LLZO phase (**Figure** **3**a). Whereas, in 5.0 wt% LLZO‐PAN/LiClO_4_ composite electrolyte, the Li ions prefer to transport through the LLZO‐modified PAN phase rather than the unmodified PAN regions according to the results of solid‐state ^6^Li NMR (Figure 3b).^52^ Zheng and Hu^64^ also examined the effects of active filler concentration on Li ion transport pathways in LLZO‐P(EO)_18_/LiTFSI system (Figure 3c). It is found that with the increase in the fraction of LLZO phase, the main ion transport pathway shifts from polymer to ceramic phase. The Li ion transport pathways greatly depend on the composition and structure of SCEs. When the content of ceramic filler is slight, the Li ions prefer to migrate through polymer phase or the modified interface between polymer and ceramic. As the amount of filler increases, the prior conductive path transfers to the ceramic phase due to the formation of continuous ionic conductive network by ceramic fillers. Li et al.^65^ observed the space charge region (≈3 nm) by transmission electron microscope (TEM) at the Ga‐LLZO/PEO interface. The conduction model demonstrated that the enhanced ionic conductivity could be ascribed to the ionic conduction in the space charge regions and the percolation of the space charge regions.^65^ In addition, combining the calculation and experimental results, a similar behavior has been found in the LATP/PEO composite electrolyte as well.^66^ The LATP in PEO could establish low‐energy barrier hopping channels along the surface for Li ion migration. The inherent ionic conductivity of the interphase between PEO and LATP is three to four times higher than that of PEO/LiClO_4_ with 10 wt% LATP nanoparticles, while at high LATP nanoparticle loading, the Li conduction mainly depends on direct contact between nanoparticles. However, recently, Zagórski et al.^67^ found that the polymer phase highly contributes to the long‐range Li ion transport rather than the ion‐conducting Li_6.55_Ga_0.15_La_3_Zr_2_O_12_ particles, and the polymer is constrained between ceramic particles at high filler loading (above 40 vol%), leading to restricted chain mobility and thus limited ionic conduction. In sum, as shown in **Table** **1**, the Li ions in SCEs may transport through polymer phase, ceramic bulk phase, and the interface region between polymer and ceramic, which is greatly depended on the types, content, and morphology of ICEs. Furthermore, the interphase between polymer and ceramic filler undoubtedly creates fast transport channel for lithium ion. Constructing a continuous high‐efficient Li ion transport channel with sufficient interphase could greatly improve the ionic conductivity of SCEs. Therefore, it seems quite necessary and significant to regulate the morphology of ceramic fillers embedded in SCEs.

**Table 1:** The conclusion summary of different researches focused on the mechanism of Li‐ion migration in SCEs

Composition	Method	Conclusion	Refs.
50 wt% LLZO‐PEO_18_/LiClO_4_	Selective isotope labeling and high‐resolution solid‐state Li NMR	Li ions preferentially move through the Li_7_La_3_Zr_2_O_12_ ceramic phase.	62
5 wt% LLZO nanowires‐PAN/ LiClO_4_	Selective isotope labeling and high‐resolution solid‐state Li NMR	Li ions preferentially move through the modified regions at the LLZO/polymer interface.	52
5 wt% LLZO‐PEO_18_/LiTFSI	Selective isotope labeling and high‐resolution solid‐state Li NMR	Li ions preferentially move through the polymer phase.	64
20 wt% LLZO‐PEO_18_/LiTFSI		Li ions preferentially move through the LiTFSI dispersed in PEO and partially from decomposed LLZO.	
50 wt% LLZO‐PEO_18_/LiTFSI		Li ions preferentially move through the percolated network formed by LLZO particles.	
50 wt% LLZO‐PEO_18_/LiTFSI‐(50 wt%)‐TEGDME		Li ions preferentially move through the PEO−TEGDME matrix.	
16 vol% Ga‐LLZO‐PEO	The ionic conductivity data and Monte Carlo simulation	The space charge region is observed and the enhanced ionic conductivity can be ascribed to the space charge region	65
10 wt% LATP‐PEO/LiClO_4_	The ionic conductivity data and simulation and transmission electron microscope	The enhanced ionic conductivity can be ascribed to the interphase region surrounding the particles, which achieves percolation at low nanoparticle loading.	66
70 wt% LLZO‐P(EO)_15_/LiTFSI	The ionic conductivity data	Li ions are trapped at the interface and/or within the LLZO surface layer, which depletes the ionic conductivity.	53

### 2.2 Effects of Ceramic Morphology on Ionic Conductivity of SCEs

According to the above results of ion‐conducting mechanism in SCEs, Li ions tend to transfer along interface layer between polymer and ceramic.^40^, ^59^ Therefore, the morphology of ceramics and especially the interface design doubtlessly plays important roles in enhancing the ionic conductivity of SCEs.^68^ The effects of ceramic morphology including particle size, shape, and arrangement on ionic conductivity of SCEs are discussed in the following content.

#### 2.2.1 Particle Size of Ceramic Fillers

**Figure 4:**
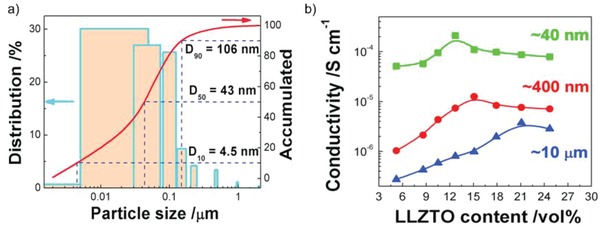
a) Size distribution of the LLZTO nanoparticles determined by a laser particle size analyzer. *D*_10_ of 4.5 nm, *D*_50_ of 43 nm, and *D*_90_ of 106 nm are evaluated according to the reference. b) The conductivity as a function of LLZTO volume fraction of the LLZTO particles with different sizes. Reproduced with permission.^47^ Copyright 2016, Elsevier.

The particle size of embedded ceramics has a significant effect on the ionic conductivity of SCEs. LAGP with different particle sizes was applied to modify the P(EO)_18_/LiTFSI electrolyte. The results show that the P(EO)_18_/LiTFSI solid electrolyte with 20% of the smallest LAGP particles (smaller than 500 nm) exhibits a maximum ionic conductivity of 6.76 × 10^−4^ S cm^−1^ and an electrochemical window up to 5.3 V versus Li^+^/Li at 60 °C.^38^ In addition, Zhang et al.^47^ presented that the Li‐salt‐free PEO with ≈40 nm LLZTO particles showed an ionic conductivity of 2.1 × 10^−4^ S cm^−1^ at 30 °C, which is much higher than those of PEO with ≈10 µm and ≈400 nm LLZTO particles (3.8 × 10^−6^ and 1.3 × 10^−5^ S cm^−1^, respectively) (**Figure** **4**). Based on the previous results, it can be concluded that the smaller particle with larger specific surface area and more abundant active sites could decrease the crystalline of polymer hosts and promote the dissociation of lithium salts more effectively, and thus provide more ion‐conducting highways for Li ions. Recently, Sun et al.^69^ mixed bimodal‐sized Li_7_La_3_Zr_2_O_12_ fillers into PVDF/LiClO_4_ polymer electrolyte (X‐CPE), resulting in an ionic conductivity of 2.6 × 10^−4^ S cm^−1^ at room temperature. Further characterization indicates that densely packed LLZO fillers with nano‐ and micro‐meter size contribute to the formation of long‐range Li ion pathways and thus high ionic conductivity. Obviously, the nano‐sized particles facilitate the ionic conductivity enhancement of SCEs and the smaller size produces the higher ionic conductivity. However, the ceramic nanoparticles are prone to agglomeration and phase separation in polymer matrix due to their high surface energy. In order to achieve high homogeneity of SCEs and avoid nonuniform Li ion transportation, some special shapes of ceramic fillers are developed to reduce the agglomeration as shown in the following content.

#### 2.2.2 Shape of Ceramic Fillers

**Figure 5:**
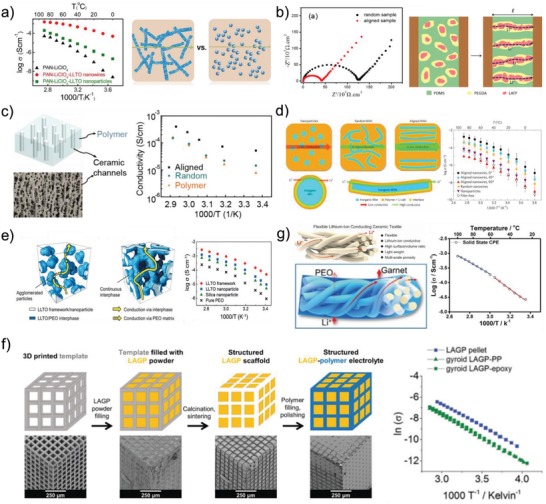
a) Dependence of ionic conductivity of PAN/LiClO_4_, PAN/LiClO_4_ with LLTO nanowires, and LLTO nanoparticles on temperature. Reproduced with permission.^40^ Copyright 2015, American Chemical Society. b) Illustration of electric‐field‐directed parallel alignment of LATP@PEGDA, resulting in improved ion conductivity. The electric‐field direction is indicated by the parallel arrow. Reproduced with permission.^76^ Copyright 2018, American Chemical Society. c) Schematic of vertically aligned and connected ceramic channels for enhancing ionic conduction and ionic conductivities of the three structures, the pure PEO/PEG, PEO/PEG/randomly dispersed LATP nanoparticles, and PEO/PEG/ice‐templated LATP nanoparticles electrolytes, at different temperatures. Reproduced with permission.^60^ Copyright 2017, American Chemical Society. d) The comparison of possible Li‐ion conduction pathways. Arrhenius plots of the composite polymer electrolytes with aligned nanowire arrays at various orientations, together with the data for the composite electrolyte with randomly dispersed nanowires and the filler‐free electrolyte. Reproduced with permission.^59^ Copyright 2017, Elsevier. e) Schematic representation of possible conduction mechanism in composite electrolytes with agglomerated nanoparticles and 3D continuous framework. Ionic conductivity of LLTO framework, LLTO nanoparticle, and silica particle composite electrolytes. Reproduced with permission.^55^ Copyright 2017, John Wiley and Sons. f) Schematic of SCEs produced by 3D printing technology and their Arrhenius plot of the lithium‐ion conductivity. Reproduced with permission.^80^ Copyright 2018, Royal Society of Chemistry. g) Schematic and Arrhenius plot of the lithium‐ion conductivity of flexible lithium‐ion conducting ceramic textile. Reproduced with permission.^82^ Copyright 2018, Elsevier.

Apart from particle size of ICEs, the shape of ICEs also presents significant influence on the Li ion conduction in SCEs. The direction and length of Li ion pathway provided by ICEs are directly determined by their shape. Cui and co‐workers^40^ compared the effect of LLTO nanoparticles and nanowires on the electrochemical properties of PAN/LiClO_4_ solid electrolyte. It was found that the PAN/LiClO_4_ with 15 wt% LLTO nanowires displays a much higher ionic conductivity of 2.4 × 10^−4^ S cm^−1^ at room temperature than that of PAN/LiClO_4_ and PAN‐LiClO_4_ with 15 wt% LLTO nanoparticles (2.1 × 10^−7^ and 0.5 × 10^−5^ S cm^−1^, respectively) (**Figure** **5**a). The enhancement of ionic conductivity could be mainly attributed to the construction of continuous 3D ion‐conducting network pathway, providing long‐range fast Li‐ion lithium transfer channels; whereas, the LLTO nanoparticles were isolated in polymer matrix to form discontinuous pathways. Hu and co‐workers^41^ fabricated a fiber‐reinforced polymer composite membrane (FRPC) by filling the PEO/LiTFSI hybrid into a Li_6.4_La_3_Zr_2_Al_0.2_O_12_ 3D nanofiber network. The FRPC membrane displays an ionic conductivity of 2.5 × 10^−4^ S cm^−1^ at room temperature, one or two orders of magnitude higher than that of PEO‐based electrolytes with LLZO nanoparticles.^46^, ^50^, ^53^ The symmetric Li/FRPC/Li cell could steadily cycle for about 500 h at a current density of 0.2 mA cm^−2^ and over 300 h with a current density of 0.5 mA cm^−2^. Recently, Wang et al.^70^ developed a novel lithium‐salt‐rich PEO/Li_0.3_La_0.557_TiO_3_ interpenetrating SCEs with 3D ceramic nano‐backbone by hot‐pressing and quenching, in which the concentration of LiTFSI reaches 40 wt%. This SCEs achieves an ionic conductivity of 1.8 × 10^−4^ S cm^−1^ at room temperature and an electrochemical window of 4.5 V versus Li/Li^+^. More importantly, the as‐prepared symmetric Li/Li cell using above novel SCEs can steadily cycle for over 800 h at a current density of 0.1 mA cm^−2^. Furthermore, some other researches[qv: 24b,71–73,104] also demonstrated that the ceramic nanowires can really enhance the Li ion conduction in polymer electrolyte. Except nanowire, the ceramic nanosheet also plays a similar role that offers continuous and high‐efficient ion‐conducting pathway. Song et al.^74^ fabricated Li_6.5_La_3_Zr_1.5_Nb_0.5_O_12_ nanosheets with GO as a template and then developed the PEO‐based composite electrolyte with 15 wt% Li_6.5_La_3_Zr_1.5_Nb_0.5_O_12_ nanosheets, which exhibits a conductivity of 3.6 × 10^−4^ S cm^−1^ at room temperature. Moreover, the mesoporous LiAlO_4_ nanosheets were filled into P(EO)_16_/LiClO_4_ polymer matrix to fabricate SCEs with enhanced ionic conduction and transfer efficiency.^75^ Therefore, compared to the nanoparticles of ceramic fillers, the nanowires, fiber, and nanosheets in polymer host are able to provide continuous Li ion transport channels and thus higher ionic conductivity could be obtained. In addition, the nanowire, fiber, and nanosheet of ceramic fillers can construct the 3D or 2D Li ion transport channels and network for high‐efficient long‐range Li ion transportation.

#### 2.2.3 Arrangement of Ceramic Fillers

Generally, 10–20 wt% of ceramic fillers is the critical addition content in SCEs. However, the severe agglomeration behavior of the particles greatly lowers the volume fraction of interphase, destroys the percolated network of interphase, and thus reduces the ionic conductivity.^55^ In addition, since the nanoparticles and nanowires are randomly mixed within polymer matrix, the Li ion transport channels constructed in SCEs are disordered. If the ceramic fillers distribute linearly along the current direction in SCEs, the migration of Li ions would become more targeted and efficient. Therefore, various methods have been adopted to obtain SCEs with aligned ceramic fillers for larger ICEs/polymer interphase and high‐efficient Li ion transportation. Liu et al.^76^ induced the LATP particles coated with poly(ethylene glycol) diacrylate (PEGDA) to distribute orderly into the poly(dimethylsiloxane) (PDMS) matrix by an external alternating‐current electric field, which architects 3D lithium‐ion conductive networks in SCE (Figure 5b). The ionic conductivity of the aligned LATP@PEGDA@PDMS is three times that of unaligned LATP@PEGDA@PDMS under the same condition. Yang co‐workers^60^, ^77^ reported an easy and simple ice‐templating‐based method to prepare PEO‐based composite electrolyte with vertically aligned and connected LATP nanoparticles. Both of the PEO and PEO/polyethylene glycol (PEG) polymer electrolytes with ice‐templated LATP nanoparticles show higher ionic conductivity than those without filler or with randomly dispersed LATP nanoparticles (Figure 5c). Furthermore, Cui co‐workers^59^ also proved the significance of alignment of ceramic nanowires for the enhancement of ionic conductivity of SCEs, including the angles among the nanowires. They embedded the aligned LLTO nanowires in PAN‐LiClO_4_ polymer host with various orientations to fabricate SCEs. The ionic conductivity of the PAN‐LiClO_4_ electrolyte with randomly dispersed LLTO nanowires at 30 °C is 5.40 × 10^−6^ S cm^−1^. Whereas, for the case of nanowires aligned at 90°, 45°, and 0°, the ionic conductivities are 1.78 × 10^−7^, 2.24 × 10^−5^, and 6.05 × 10^−5^ S cm^−1^, respectively. The activation energies for electrolyte with random nanowires and electrolytes with 0° aligned nanowires are respectively 0.84 ± 0.02 and 0.94 ± 0.04 eV, indicating faster ion conductive pathway contributed by the aligned nanowires. The ion‐conducting pathway without crossing junctions can further improve the ionic transportation efficiency (Figure 5d). Therefore, the significant enhancement of ionic conductivity of SCEs with aligned ceramic nanoparticles and nanowires is attributed to the continuous and aligned lithium‐ion conductive pathways facilitating the lithium‐ion motion in SCEs. In addition, the alignment of the ceramic could further shorten the transfer distance of Li ions and enhance the ionic conductivity of SCEs.

It is also found that the 3D ceramic framework not only greatly augments the continuous and integrated ion‐conductive network, but also increases the mechanical strength of SCEs. Bae et al.^55^ proposed a 3D hydrogel‐derived nanostructured Li_0.35_La_0.55_TiO_3_ (LLTO) framework as high‐loaded nanofillers of SCE. The interconnected structure of 3D LLTO framework provides a long‐range and continuous Li ion pathway resulting in an ionic conductivity of 8.8 × 10^−5^ S cm^−1^ at room temperature (Figure 5e) and endows with a high flexibility of SCE. The similar results are also obtained in the SCE with 3D nanostructured garnet frameworks.^56^ Xie et al.^78^ developed another strategy for the preparation of hybrid electrolyte membrane with a cubic LLZO interconnected network using bacteria cellulose (BC) as a template and PEO‐LiTFSI polymer. The well‐organized LLZO network also constructs long transport pathways for Li ion motion and thus enables the SCE to exhibit an elevated conductivity of 1.12 × 10^−4^ S cm^−1^. Similar alignment strategy could be employed to the SCE with 3D ion‐conductive ceramic framework as well. For example, Hu co‐workers^79^ fabricated mesoporous aligned garnet nanostructure using wood as template where the ion‐conductive PEO was cast into the aligned garnet to prepare the SCE. This SCE presents both a high ionic conductivity (1.8 × 10^−4^ S cm^−1^ at room temperature) and good mechanical flexibility.

Furthermore, the ordered 3D ceramic electrolytes were also prepared by 3D printing technology as an effective method to construct 3D ceramic framework. For instance, Zekoll et al.^80^ constructed 3D ordered ceramic electrolyte (LAGP) with bicontinuous microchannels in polymers such as epoxy polymer and polypropylene using 3D printing templates with ordered cubic, gyroidal, diamond‐shaped structure as well as bijel‐derived microarchitecture (Figure 5f). The SCE with gyroidal architecture presents a total ionic conductivity of 1.6 × 10^−4^ S cm^−1^ at room temperature, only slightly lower than a dense ceramic pellet with 2.8 × 10^−4^ S cm^−1^. Moreover, the gyroidal hybrid electrolyte has much higher mechanical strength than pure LAGP pellet, and presents a better restriction for lithium dendrite growth. Therefore, the SCEs with well‐integrated structure could effectively enhance Li ion conduction and effectively prevent the agglomeration of fillers even at high concentration.

Except for the 3D ceramic framework, the ceramic fabrics manufactured by electrostatic spinning and template synthesis are another commendable reinforcement to improve the performance of SCEs, which endow the SCEs with high mechanical strength and ionic conductivity. Since the nanofibers could effectively provide long‐range and smooth conduction pathways for Li‐ions, well aligned Li_6.4_La_3_Zr_2_Al_0.2_O_12_ nanofiber films were embedded in elastic ionic‐conductive PVDF polymer to form SCE, which shows an ionic conductivity of 1.12 × 10^−4^ S cm^−1^ at 30 °C with a low activation energy of 0.308 eV.^81^ Hu and co‐workers^82^ composited the garnet‐based lithium‐ion conductive ceramic textiles derived from the cellulose textile template method with PEO‐based polymer electrolyte (Figure 5g), which demonstrated high lithium‐ion conductivities of 2.7 × 10^−5^ S cm^−1^ at 25 °C and 1.8 × 10^−4^ S cm^−1^ at 60 °C. The Li ion migration along the fibers enhances the lithium ions transfer, which makes the SCEs less dependence on the solvated lithium content.

**Figure 6:**
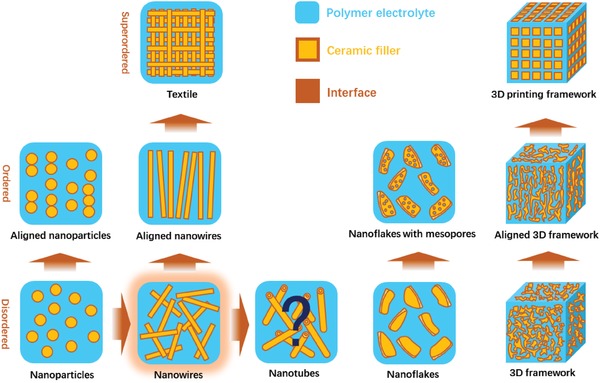
The evolutions of morphology of ceramic filler in SCEs.

In general, although the SCEs exhibit enhanced ionic conductivity, the total ionic conductivity is limited by polymer matrix. There are still many challenges to achieve acceptable value for practical application of SCEs at room temperature. In order to enhance the ionic conductivity of SCEs, the ceramic fillers with different morphologies are introduced to reduce the crystallinity of polymer electrolyte and construct high‐efficient ionic transportation channels.^83^ The ceramic fillers were developed from isolated 0D particles, 1D nanowires, and 2D nanosheets to continuous 3D frameworks and bulk components (**Figure** **6**).^15^, ^16^, ^84^ Among all the ceramics with different morphologies, the nanowires show great improvement on the ionic conductivity of polymer electrolytes as shown in Figure 2 and **Table** **2**. Although, the 3D framework possesses more continuous conductive pathway for the migration of lithium‐ions, large number of voids in SCEs may lead to incompatible and poorly contacted interfaces, which hinders the lithium‐ion transfer to some content. In the most of the present research work, the electrochemical performance data of as‐prepared lithium batteries were obtained at high temperature (beyond glass transition temperature of polymer matrix). Thus, in order to enable application of SCEs at room temperature, their ionic conductivity still needs to be further improved by structure design of ceramic and composition tuning of polymer matrix.

**Table 2:** Electrochemical performance of SCEs with different ceramic fillers and morphologies

Morphology	Alignment	Ceramic filler	Polymer host	Percolation	Ionic conductivity	Electrochemical window (vs Li/Li^+^)	Refs.
Nanoparticle	No	Li_6.75_La_3_Zr_1.75_Nb_0.25_O_12_	PVDF/LiClO_4_ (weight ratio = 3: 1)	7.5 wt%	9.2 × 10^−5^ S cm^−1^ at 25 °C	4.6 V	85
	No	LAGP	polyethylene terephthalate/poly(ionic liquid)/LiTFSI	10 wt%	7.78 × 10^−5^ S cm^−1^ at 30 °C	4.55 V	86
	No	LATP@PEGDA	poly(dimethylsiloxane)		2.4 × 10^−6^ S cm^−1^ at 25 °C		76
	Yes				8.0 × 10^−7^ S cm^−1^ at 25 °C		
	Yes	LAGP	PEO:PEG = 1:1, LiClO_4_, [O]:Li^+^ = 8:1		1.67 × 10^−4^ S cm^−1^ at 25 °C		77
	Yes	LATP	PEG:PEO = 1:1.15, LiClO_4_, [O]:Li^+^ = 8:1		5.2 × 10^−5^ S cm^−1^ at 25 °C		60
Nanowire	No	LLTO	PEO/LiTFSI (weight ratio = 2: 1)	15 wt%	2.4 × 10^−4^ S cm^−1^ at 25 °C	5 V	72
	No	LLTO	PAN/LiClO_4_	15 wt%	2.4 × 10^−5^ S cm^−1^ at 25 °C		40
	No	Li_6.4_La_3_Zr_2_Al_0.2_O_12_	PEO/LiTFSI		2.5 × 10^−4^ S cm^−1^ at 25 °C	6.0 V	41
	No	Li_6.75_La_3_Zr_1.75_Nb_0.25_O_12_	poly(methyl methacrylate)/LiClO_4_	10 wt%	2.2 × 10^−5^ S cm^−1^ at 25 °C	5.5 V	73
	No	LLZO	PVDF‐HFP/LiTFSI	10 wt%	9.5 × 10^−4^ S cm^−1^ at 25 °C	4.7 V	87
	Yes	LLTO	PAN/LiClO_4_		6.05 × 10^−5^ S cm^−1^ at 30 °C		59
Nanosheet	No	Li_6.5_La_3_Zr_1.5_Nb_0.5_O_12_	P(EO)_10_/LiClO_4_	15 wt%	3.6 × 10^−4^ S cm^−1^ at 25 °C		74
Fabric	No	LLTO	PEO with 40 wt% LiTFSI		1.8 × 10^−4^ S cm^−1^ at 25 °C	4.5 V	70
	Yes	Li_6.28_Al_0.24_La_3_Zr_2_O_11.98_	PEO/LiTFSI		2.7 × 10^−5^ S cm^−1^ at 25 °C		82
	Yes	Li_6.4_La_3_Zr_2_Al_0.2_O_12_	PVDF/LiClO_4_ (weight ratio = 3:1)		1.16 × 10^−4^ S cm^−1^ at 30 °C	5.0 V	81
3D framework	No	Ga‐LLZO	P(EO)_12_/LiTFSI		1.2 × 10^−4^ S cm^−1^ at 30 °C	5.6 V	88
	No	LLZO	PEO/LiTFSI (weight ratio = 2:1)		8.9 × 10^−5^ S cm^−1^ at 25 °C	5.5 V	22
	No	LLZO	PEO/LiTFSI		1.14 × 10^−4^ S cm^−1^ at 25 °C	6.0 V	78
	No	LLTO	P(EO)_10_/LiTFSI		8.8 × 10^−5^ S cm^−1^ at 25 °C	4.5 V	55
	No	Li_6.28_La_3_Zr_2_Al_0.24_O_12_	P(EO)_10_/LiTFSI		8.5 × 10^−5^ S cm^−1^ at 25 °C	5.0 V	56
	Yes	Li_6.4_La_3_Zr_2_Al_0.2_O_12_	P(EO)_8_/LiTFSI with 15 wt% succinonitrile		1.8 × 10^−4^ S cm^−1^ at 25 °C	6 V	79

## 3 Interfacial Issues and Remedies in All‐Solid‐State Battery with SCEs

**Figure 7:**
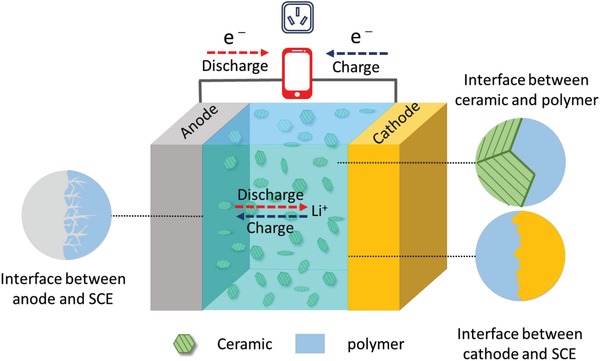
Interfaces in all‐solid‐state lithium metal battery assembled with SCE.

Generally, there are three types of interface in the as‐assembled battery with SCEs, including the interface between anode and SCEs, the interface between cathode and SCEs, and the interface between ceramic and polymer (**Figure** **7**).^36^ The poor interface between anode and SCEs usually brings about uncontrollable growth of lithium dendrite.^1^, ^89^, ^90^ Meanwhile, the volume change of electrode is inevitably accompanied with charge/discharge process, causing the contact loss of electrode with SCEs and thus large impedance.^91^ Furthermore, phase boundaries between polymer and ceramic fillers could increase the migration barrier for Li ions transportation, which results in high interfacial impedance and poor electrochemical performance of the batteries.^10^ Therefore, in order to tackle the poor contact and low conduction commonly at the interfaces, structure construction of SCEs and surface modification are strongly required.^92^, ^93^

### 3.1 Interfacial Issues and Remedies between SCE and Lithium Anode

**Figure 8:**
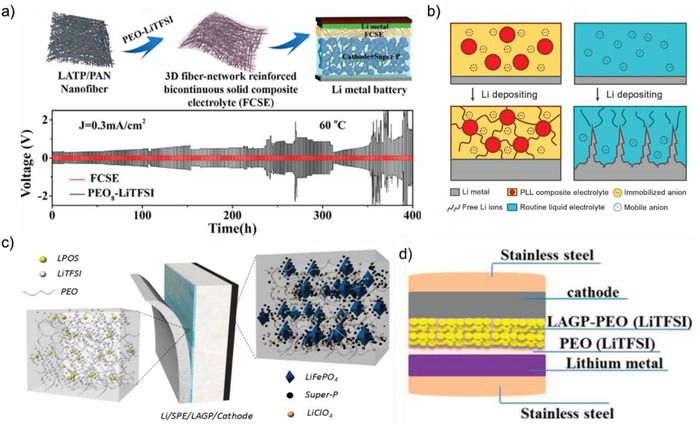
a) Schematic illustration for the preparation of FCSE. Reproduced with permission.^97^ Copyright 2018, American Chemical Society. b) Schematic of the electrochemical deposition behavior of the Li metal anode with the PLL solid electrolyte with immobilized anions and the routine liquid electrolyte with mobile anions. Reproduced with permission.^50^ Copyright 2017, PNAS. c) Schematic of the ASSLB with optimized cell structure. Reproduced with permission.^98^ Copyright 2017, Royal Society of Chemistry. d) All‐solid‐state Li/PEO/LAGP‐PEO/LiMFP cells. Reproduced with permission.^99^ Copyright 2017, American Chemical Society.

The poor contact between Li anode and ICEs induces the high impedance and heterogeneous Li ion deposition that causes the inferior rate performance and uncontrollable lithium dendrite growth.^94^, ^95^ Inheriting the advantages of both SPEs and ICEs, the SCEs show good stability with Li metal and satisfactory performance in the suppression of lithium dendrite growth. The polymer hosts not only provide continuous ion‐conducting pathway and isolate ceramic particles from electrodes to avoid side reactions, but also offer soft contact with electrodes to reduce Li ion transfer resistance, which could achieve a uniform Li ion flux to suppress lithium dendrite growth.^47^, ^50^ Meanwhile, the rigid ceramic particles offer fast highways for the migration of lithium ions and enhance the mechanical strength of the electrolyte to suppress lithium dendrites.^40^, ^51^, ^96^ For instance, Zhao et al.^43^ incorporated slight LGPS into P(EO)_18_/LiTFSI matrix to fabricate a contact amelioration SCE membrane. The interfacial resistance of symmetrical cell assembled with 1% LGPS‐P(EO)_18_/LiTFSI membrane is 26 Ω cm^2^ and remains constant after storage for 7 days, while the interfacial resistance of P(EO)_18_/LiTFSI membrane obviously increases from 38 to 80 Ω cm^2^ at the same condition. The LiFePO_4_/Li cell using the incorporated membrane also shows excellent cycling performance (capacity retention of 92.5% after 50 cycles at 0.5 C rate and 60 °C) and good rate property. The interfacial resistance remains steady around 113 Ω after 20 cycles, while that of LiFePO_4_/P(EO)_18_/LiTFSI/Li increases from 203 to 270 Ω. In addition, the ceramic fillers could also reinforce the mechanical properties of the solid electrolyte to inhibit lithium dendrite growths. An SCE with high mechanical property was obtained by reinforcing the P(EO)_8_/LiTFSI polymer substrate with LATP/PAN fiber (FCSE) (**Figure** **8**a), which has an enlarged tensile strength (10.72 MPa).^97^ The Li/Li cell with FCSE exhibits excellent stability in both bulk and interface resistance after 12 days.

Since the uncontrollable growth of lithium dendrite could be ascribed to the inhomogeneous Li‐ion flux and deposition, it is of great significance to develop SCEs with uniform Li ion transportation channels for a homogeneous Li deposition. Zhang co‐workers^50^ designed an anion‐immobilized composite electrolyte based on P(EO)_18_/LiTFSI with Al‐doped LLZTO powders (PLL) (Figure 8b). Since the anions are immobilized, it is much easier for Li ions to diffuse from the bulk electrolyte to the anode surface that establishes a relative stable and uniform interface for Li ion deposition, which can effectively suppress the lithium dendrite growth. The Li/Li symmetrical cell can steadily cycle for more than 400 h with a constant voltage polarization of 15 mV at 60 °C and a current density of 0.10 mA cm^−2^.

Furthermore, the structure design of SCEs plays a significant role for strong inhibition of the lithium dendrite growth. For instance, an SCE consisting of rigid LAGP pellet and soft PEO‐1%‐75%Li_2_S·24%P_2_S_5_·1%P_2_O_5_ (LPOS) layer (ASSLB) was fabricated, which presents excellent chemical stability against the lithium metal and great inhibition to lithium dendrite growth (Figure 8c).^98^ The polarization voltage of the Li/Li cell with the double‐layer SCE could remain constant for more than 1000 h with 0.1mA cm^−2^ current density at 60 °C, indicating that the interface between SCE and lithium metal maintains stably during the long‐period test. Wang et al.^99^ adopted similar construction strategy to develop another SCE consisting of LAGP‐PEO layer and P(EO)_8_/LiTFSI layer (≈100 nm) (Figure 8d). The quite less resistance offers sufficient ionic conduction and high mechanical module, which successfully protects the Li metal from reacting with LAGP and suppresses the lithium dendrite nucleation due to excellent interfacial contact with Li metal. Therefore, the PEO‐polymer coating layer on the LAGP based SCE can effectively reduce the interfacial impedance and homogenize the Li‐ion flux to suppress Li dendrite formation, which also can prevent the side reaction between LAGP and Li metal anode.

From the above analysis, it can be obtained that the interface between lithium anode and solid‐state electrolyte is quite important for achieving the outstanding performance all‐solid‐state batteries. The SCEs not only promotes the construction of a low‐resistance interface with Li metal anode, but also can effectively suppress the Li dendrite growth and side reactions of ICEs with Li metal anode. Whereas, it should be noted that the thickness of above developed SCEs is still larger than commercial separator and the applied current density and areal capacity of plating and stripping during long cycling to examine roles for suppression of Li dendrite growth is quite lower. As we know, it is really difficult to maintain the interface stability and suppress the Li dendrite growth in all‐solid‐state batteries at a higher current density and larger plating and stripping capacity. Therefore, it is quite necessary to develop the SCEs with much thinner thickness and high mechanical strength to suppress the Li dendrite growth at larger charge/discharge areal capacity under high current density in the future research.

### 3.2 Interfacial Issues and Remedies between SCE and Cathode

The optimization of interface between cathode and SCE is another important research hotspot for the practical application of the all‐solid‐state lithium battery.^94^ Similar to the interface at the anode side, solid–solid contact exists between cathode materials and solid electrolyte, which leads to large interfacial impedance. Besides, since the cathode is low ion‐electron‐conducting continuum, constructing mixed conducting network in cathode is quite significant to achieve high electrochemical performance of all‐solid‐state lithium battery, especially at a high cathode material loading.

**Figure 9:**
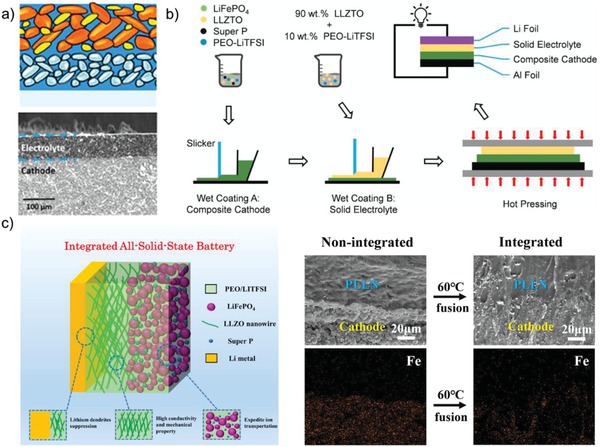
a) Schematic illustration of the interface between composite cathode containing 15 wt% polymer and the composite electrolyte. Reproduced with permission.^100^ Copyright 2017, American Chemical Society. b) Schematic illustration of the synthesis procedure by hot pressing. Reproduced with permission.^101^ Copyright 2019, Elsevier. c) Schematic illustration of an integrated all‐solid‐state LiFPO_4_/PLLN/Li battery. Reproduced with permission.^57^ Copyright 2018, John Wiley and Sons.

In order to reduce the interfacial impedance between cathode and SCEs, special structures of cathode and battery were designed. For example, Chen et al.^100^ stacked the composite LiFePO_4_ cathode, PEO filled with Al‐LLZTO particles, and lithium foil layer by layer and then hot‐pressed to form a monolithic all‐solid‐state battery (**Figure** **9**a). Ascribed to the relatively high viscosity and ductility of both composite cathode and electrolyte, no pores and voids were observed in the interfacial regions, which contribute to tight contact of cathode with SCE and quite less interfacial resistance. The as‐prepared all‐solid‐state battery demonstrates an ultrahigh surface discharge capacity of 10.8 mAh cm^−2^ and an average specific discharge capacity of 155 mAh g^−1^ at the current density of 100 µA cm^−2^ at 60 °C. Similar method was also employed by Zha et al.^101^ to fabricate all‐solid‐state Li/LiFePO_4_ battery with 90 wt% LLZTO/PEO electrolyte (Figure 9b). The interfacial resistance of the cathode/electrolyte largely reduces from ≈248 to ≈62 Ω cm^−2^ after wet coating and hot pressing. Our group reported a low resistance‐integrated all‐solid‐state battery using PEO‐based electrolyte embedded with LLZO nanowires (PLLN) and PEO/LITFSI as cathode binder (Figure 9c).^57^ The PEO in both cathode and PLLN are fused at high temperature to form a stable integrated all‐solid‐state battery structure for high‐efficient ion transportation. The integrated LiFePO_4_/Li cell presents a specific capacity of 158.8 mAh g^−1^ after 70 cycles under 0.5 C at 60 °C and a specific capacity of 158.7 mAh g^−1^ after 80 cycles at 0.1 C and 45 °C. Therefore, the monolithic and integrated structures can greatly improve structure stability and reduce the interfacial resistance of the all‐solid‐state battery, which are promising all‐solid‐state battery structures for future practical application.

In pursuit of high energy density and long cycle life of all‐solid‐state battery, the migration of electron and ion inside cathode is as crucial as the cathode/electrolyte interface.^45^, ^48^, ^57^, ^82^, ^101^, ^102^, ^103^, ^104^ Constructing ion/electron conductive networks in both the cathode and interface is of great significance for reducing the polarization and delivering full capacity of the battery. Tao et al.^48^ developed LLZO nanoparticle‐decorated porous carbon foam (LLZO@C) that was used as sulfur host. All‐solid‐state Li‐S battery was assembled using the LLZO@C/S as cathode, Li metal as anode, and LLZO‐P(EO)_8_/LiClO_4_ as both electrolyte and cathode binder. The LLZO‐P(EO)_8_/LiClO_4_ electrolyte constructs high‐efficient ionic transportation path among the sulfur particles, which can greatly reduce interfacial resistance between sulfur and LLZO‐P(EO)_8_/LiClO_4_, and enable the as‐assembled all‐solid‐state Li‐S battery to present remarkable cycling performance at 37 °C. Moreover, Zhu et al.^103^ reported a flexible electrolyte/cathode bilayer framework consisting of a 3D carbon nanofiber/sulfur (CNF/S) cathode and 1D LLTO nanofiber‐PEO electrolyte. A mixed conducting behavior was achieved by infiltrating the LLTO‐PEO solid electrolyte onto the surface and into the pores of CNF/S nanofiber membranes, thus this bilayer framework demonstrated outstanding cycle performance with high coulombic efficiency of over 99% at room temperature.

Except good interfacial contact and well‐developed mixed conducting network, the wide electrochemical window of SCEs is indispensable for constructing a stable interface between cathode and SCE, which is quite significant for the application of high‐voltage cathode in high energy density system. Generally, the electrochemical window of polymer electrolyte is below 5 V versus Li/Li^+^, while that of ceramic electrolyte could be up to 9 V or even higher.^9^, ^11^ For SCEs, the ceramic filler could widen the electrochemical window beyond 5 V to satisfy the most cathode materials with high working voltage.^38^, ^43^, ^46^, ^50^, ^54^ Generally, the larger ceramic content could result in higher electrochemical stability.^20^, ^46^, ^54^ The enhancement of electrochemical stability might be attributed to the following reasons. First, the dipole–dipole interaction between polymer chains and ceramic fillers can elevate the oxidation decomposition potential of polymer.^99^ Second, the acidic surface sites of ceramic complex with the salt anions can suppress the migration of anions and thus limit their irreversible oxidation.^38^, ^42^ Third, the inorganic ceramic fillers can remove the impurities such as water in SCE from the interface.^47^

Compared with Li metal anode, the interfacial issues at cathode side are more complex and there are exceptional challenges. Unlike the batteries using liquid electrolytes that contain mainly liquid/solid interface with quite smaller resisitance, there are numerous solid/solid interfaces with very large resisitance between cathode particles and solid electrolyte. In addition, these interfaces are unstable during long cycling of all‐solid‐state battery. Therefore, constructing high‐efficient and stable ion‐conducting networks inside the cathode is essential for practical application of all‐solid‐state battery.^105^ Special structure design of SCE and all‐solid‐state batteries such as layer‐by‐layer and integrated structure could tighten the interphase connection and reinforce the ion conduction to obtain an effective conductive network. In addition, the oxidation potential of polymer electrolyte is lower, which is prone to be oxidized and decomposed, especially coupled with high‐voltage cathode materials. Hence, it is quite significant to develop the high‐voltage‐resistant polymer matrix or improve the electrochemical window of polymer electrolyte using ICEs to broaden the SCE application in all‐solid‐state battery in future.^106^

### 3.3 Interfacial Issues and Remedies of SCEs with Both Li Anode and Cathode

**Figure 10:**
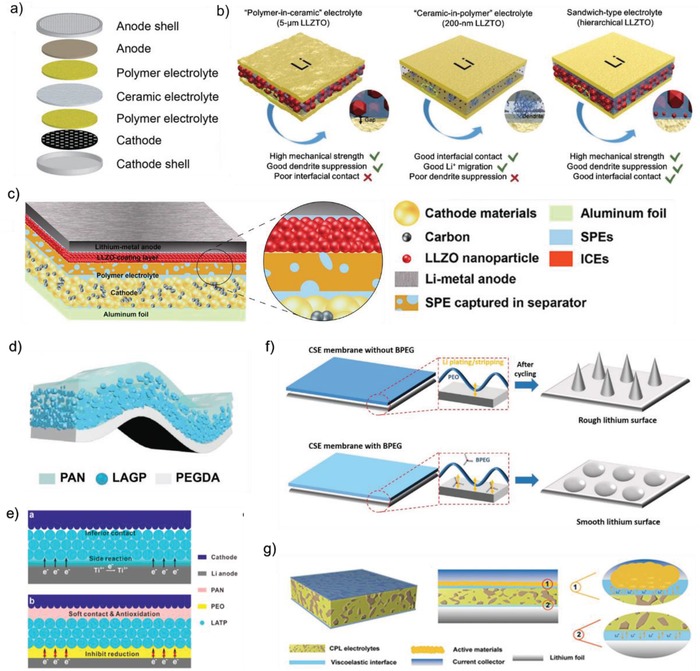
a) Illustration of all‐solid‐state battery design with the PCPSE electrolyte and the structure of polymer adopted. Reproduced with permission.^108^ Copyright 2016, American Chemical Society. b) The schematic illustration of the PIC‐5 µm, CIP‐200 nm, and hierarchical sandwich‐type composite electrolytes. Reproduced with permission.^109^ Copyright 2019, John Wiley and Sons. c–e) Three different SCEs with unsymmetrical structure fabricated by Guo et al. Reproduced with permission.^113^, ^114^, ^115^ Copyright 2018 and 2019, American Chemical Society. Copyright 2019, John Wiley and Sons. f) Proposed lithium plating/stripping processes and lithium surfaces when lithium metal is in contact with PEO‐LATP and PEO‐LATP‐BPEG membrane. Reproduced with permission.^39^ Copyright 2017, John Wiley and Sons. g) The schematic diagram of CPL composite electrolyte with viscoelastic ionic liquid interface layer. Reproduced with permission.^116^ Copyright 2019, John Wiley and Sons.

Above discussion indicates that the interfacial issues between SCEs and Li anode or cathode such as large resistance and unstable interface can be effectively solved by special design of SCEs and its battery structure. In addition, construction of effective Li‐ion pathway and tight contact interface with both cathode and anode is strongly required for preparing the all‐solid‐state battery.^92^, ^107^ There are some works to simultaneously solve the interfacial issues between SCEs and Li anode and cathode by designing the SCEs with unique structure and components. Goodenough and co‐workers^108^ first proposed the concept of polymer/ceramic/polymer sandwich electrolyte (PCPSE) (**Figure** **10**a). On one hand, the introduction of ceramic layer blocks the anions of the polymer salt and thus reduces the double‐layer electric field at the Li/polymer interface, which reduces the chemical/electrochemical decomposition of the polymer electrolyte and improves the coulombic efficiency of the battery. On the other hand, the polymer layers provide wet contact with both LiFePO_4_ cathode and Li metal anode, which leads to lower Li ion transfer resistance and a uniform Li ion flux across the interface and thus suppress the Li dendrite formation, and the Li/LiFePO_4_ cell delivering a stable capacity of 130 mAh g^−1^ after 100 cycles with an efficiency of 99.7–100%. Huo et al.^109^ also reported a sandwich‐type SCE consisting of a “polymer‐in‐ceramic” interlayer of 80 vol% 5 µm LLZTO (PIC‐5 µm) sandwiched between two “ceramic‐in‐polymer” thin‐film outer layers of 20 vol% 200 nm LLZTO particles (CIP‐200 nm) (Figure 10b). The PIC‐5 µm interlayer presents high mechanical strength, which can effectively suppress the Li dendrite growth. The CIP‐200 nm layer on both anode and cathode sides possesses a smooth and flexibility surface with a high t_Li_^+^ of 0.47, which can achieve an excellent compatible contact with both Li anode and LiFePO_4_ cathode to reduce the interfacial resistance. As a result, the LiFePO_4_/SCE/Li cells present excellent cycle performance at room temperature. Some other researches also adopt similar strategy to simultaneously modify the interface of SCE with both cathode and anode.^110^, ^111^ The polymer layers provide soft contact with both electrodes and rigid layer guarantees mechanical properties.

Due to the different characteristics of cathode and anode, SCEs with Janus structure were constructed to provide more adaptable solutions for building both excellent interfaces with cathode and anode.^112^, ^113^, ^114^, ^115^ Guo and co‐workers^113^ proposed a thin asymmetric SCE with high modulus to suppress Li dendrite growth and flexibility to enable low interface resistance with both cathode and anode. In this asymmetric SCE, a rigid LLZO layer (5.7 µm) modified with an ultrathin 7.5 nm polymer is coated on one side surface of separator, which not only can effectively suppress the Li dendrite growth, but also form a stable and lower resistance interface with the Li metal anode. In addition, a soft polymer layer (5.4 µm) is used to modify the other side surface of separator, which spreads exterior and interior of cathode and separator to form connected interfaces with cathode and an integrated battery structure (Figure 10c). A continuous ion pathway is formed from anode to cathode. With this asymmetrical design, the Li/Li symmetrical cell delivers an extremely flat and stable voltage plateau for 3200 h, indicating stable lithium plating/stripping reversibility and the significant restrained Li dendrite growth. Guo and co‐workers^114^ also developed a type of heterogeneous multilayered solid electrolyte (HMSE), in which the oxidation‐resistant PAN contacts with high‐voltage cathode, in‐situ photopolymerizable reduction‐tolerant polyethylene glycol diacrylate (PEGDA) faces the Li metal anode, and a Janus and flexible PAN@LAGP (80 wt%) composite electrolyte is designed as intermediate layer (Figure 10d). The HMSE presents effective inhibition to Li dendrite growth and excellent compatibility with both cathode and anode. The Li/Li symmetric batteries using the HMSE can cycle steadily for more than 1000 h under 2 mA cm^−2^ with a stable polarization less than 40 mV. When paired with LiNi_0.6_Co_0.2_Mn_0.2_O_2_ and LiNi_0.8_Co_0.1_Mn_0.1_O_2_ cathodes, the solid‐state Li metal batteries using HMSE exhibit excellent electrochemical performance. Furthermore, Guo and co‐workers^115^ designed a compatible SCE by coating the PAN and PEO layers to both sides of LATP ceramic electrolyte (Figure 10e). The PAN‐based layer constructs soft‐contact and high‐voltage tolerance with LiNi_0.6_Mn_0.2_Co_0.2_O_2_ cathode, meanwhile the PEO layer at anode side can effectively protect the Li metal from reacting with LATP and release the electric double‐layer accumulation between LATP and Li anode to suppress Li dendrite growth. These works demonstrate the extreme significance to construct the Janus structure of SCE for high performance of all‐solid‐state lithium metal batteries. The Janus structure design for SCE can greatly enhance the mechanical strength and reduce the thickness of SCE, which is also quite significant for preparing the all‐solid‐state Li metal battery with high volumetric energy density.

Although the polymer provides tight contact with Li metal anode, direct contact between ceramics particles of SCE and Li metal anode could not be completely evicted, which would lead to potential side reaction of ceramics particles with Li metal and inhomogeneous charge deposition. Meanwhile, voids or pores may be formed between polymer chains and the surface of cathode and anode, resulting in some empty contact points and thus high impedance. Pan et al.^39^ developed a flexible SCE membrane consisting of LATP, PEO, and boronized polyethylene glycol (BPEG) (Figure 10f). They adopted small‐sized BPEG to fill those voids, which creates soft contact with Li metal with large contact area for high‐efficient lithium ion transfer. The composite membrane shows admirable capability on suppressing the formation of lithium dendrites owing to the compact and rigid LATP barrier and stable soft contacting interface induced by PEO and BPEG. Due to the enhanced ion conductivity and improved SCE/LiFePO_4_ cathode contact, the as‐fabricated solid state LiFePO_4_/Li cell presents good rate capability.

Furthermore, in order to obtain wet interfacial contact of SCE with cathode and anode, tiny liquid electrolyte or ionic liquid (less than 5 µL) was often dropped onto the interface between electrode and solid electrolyte, which can completely reduce the interfacial impedance and achieve high‐efficient ion‐transport at interface. Ma et al.^116^ developed a novel cellulose acetate/PEG/LATP (CPL) and constructed viscoelastic and nonflammable interface by adding 2.5 µL ionic liquids to both sides of CPL, which successfully solves the contact issue of CPL with both cathode and anode, sluggish kinetic process, and lithium dendrite growth (Figure 10g). The similar strategy is put forward to prepare a novel SCEs consisting of PEO, LLZTO, and slight ionic liquid ([BMIM]TF_2_N).^117^ The ionic liquid can wet the interface of SCEs with both cathode and anode and thus decreases the interface impedance while maintaining the SCEs as solid‐state membranes. The well wetted membrane electrolyte shows a conductivity of 2.2 × 10^−4^ S cm^−1^ at 20 °C approximate one order of magnitude higher than that of PEO/LLZTO. At present, tiny liquid electrolyte or ionic liquid has been widely applied in the interface between SCEs and electrodes, especially at the interface with cathode.

In sum, in order to solve the interfacial problems of anode and cathode simultaneously, it is necessary and feasible to develop multi‐layer symmetrical/asymmetric SCEs according to the characteristics of anode and cathode.^68^ These SCEs could better adapt to the interface changes in the charging and discharging process and reduce the interfacial impedance. In addition, the addition of an extremely small amount of liquid electrolyte or ionic liquid at the interface could better wet the interface and keep the properties of the solid state for batteries.

### 3.4 Interface Issues and Remedies between Ceramic and Polymer

**Figure 11:**
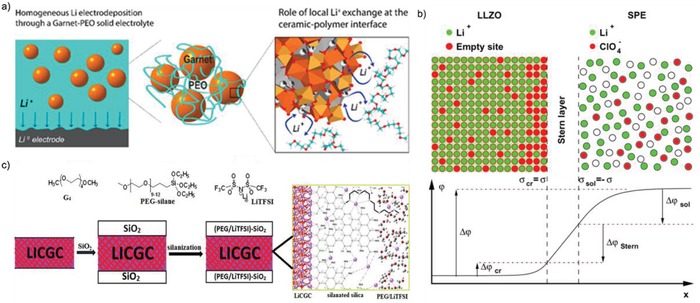
a) Schematic illustration of the interfacial Li ion exchange between LLZO particles and PEO/LiTFSI matrix. Reproduced with permission.^67^ Copyright 2019, American Chemical Society. b) The model scheme of electric double layer at the interface between LLZO and SPE. Reproduced with permission.^119^ Copyright 2019, American Chemical Society. c) Schematic of structure of SiO_2_‐coated LICGC (SiO_2_‐LICGC) functionalized with PEG‐silane in the presence of LiTFSI. Reproduced with permission.^120^ Copyright 2017, American Chemical Society.

Interface issues could not only exist between electrodes and SCEs, but also perplex the good connection between polymer chains and ceramic fillers. There have been many researches focusing on new approaches to improve the interface contact between polymers and ceramic. Whereas, the interfaces between ceramic nanoparticles and polymer chains in SCEs receive little attentions, but it is of great significance to construct an interface between ceramic and polymer with little resistance for high‐performance all‐solid‐state batteries.^27^ Constructing excellent interfaces between ceramic fillers and polymer chains in SCEs could effectively solve the intrinsic problems of SCE to achieve high‐performance all‐solid‐state batteries. Frederieke Langer et al.^118^ investigated lithium ion transition resistance of the ceramic/polymer interface using the Cu/PEO/LLZO/PEO/Cu system by electrochemical impedance spectroscopy (EIS) to identify and quantify the interface transport processes. It revealed that the activation energy of Li ion transport across the interface is as high as 0.9 eV at temperature above the melting point of PEO and lithium ion transition was impeded. Zagórski et al.^67^ presented the similar conclusion that a high interfacial resistance between the garnet particles and the PEO/LiTFSI matrix that could reach ≈10^4^ Ω cm^2^ (**Figure** **11**a). Moreover, the local Li ion exchange at the LLZO/PEO interface leads to the minimization of the Li ion concentration gradient in the SCE, which facilitates the Li ion homogenous deposition and thus avoiding Li dendrite growth. To further investigate the origin of high LLZO/polymer interfacial resistance, Brogioli et al.^119^ derived the mathematical model of the diffuse layers following the traditional derivation of the Gouy–Chapman–Stern equation (Figure 11b). By combining the theoretical calculations and experiments, it could be deduced that the large value of the interface resistance comes from high activation energy and does not from the electrostatic repulsion of lithium ion.

In order to minimize the high resistance for ion transport at the polymer/ceramic electrolyte interface, the barriers for Li ions migration across the interfaces between polymers and ceramics can be greatly reduced by modifying the ceramics. Chinnam and Wunder^120^ developed a feasible solution to address this issue. The inorganic lithium ion‐conducting glass ceramics (LICGCs) Li_2_O‐Al_2_O_3_‐SiO_2_‐P_2_O_5_‐TiO_2_‐GeO_2_ were sputter‐coated with a 200 nm thick SiO_2_ layer and then silanated with CH_3_O(CH_2_CH_2_O)_x_(CH_2_)_3_Si(OCH_2_CH_3_)_3_ (PEG‐silane) in the presence of LiTFSI (Figure 11c). After silanization, the PEG‐silane/LiTFSI shows superior contact with SiO_2_‐LICGCs, which provide crucial lithium ion‐conducting channels to considerably decrease the interfacial resistance. In addition, the ball milling at low velocity and temperature is a simple and low‐cost way to facilitate the uniform dispersion of ceramic fillers in polymer host and construct more efficient interface between ceramic and polymer.^74^, ^121^, ^122^

On account of highly ion‐conducting interphase between ceramic and polymer, the homogeneous dispersion of ceramic fillers doubtlessly contributes to the construction of continuous high‐speed and uniform Li ion migration channels.^66^ The particle size, shape, arrangement, and 3D ceramic framework of ceramic in SCEs may have significant effects on the interface properties between ceramic and polymer. Despite interface investigation in starting stage, some physical and chemical methods have been adopted to create compatible interface of ceramic with polymer, which is significant for promoting the development and application of all‐solid‐state lithium battery.

## 4 The Applications of SCEs in Lithium Metal Batteries

Based on the advantages such as high ionic conductivity, wide electrochemical windows, good interfacial contact, and effective lithium dendrite suppression, the SCEs are highly desired for application in all‐solid‐state lithium batteries. In this section, we mainly discuss the applications of SCEs in typical lithium metal batteries, Li‐S battery, and Li‐O_2_/air battery to deepen the whole comprehension of SCEs (**Table** **3**).

**Table 3:** Performance comparison of various lithium batteries with different SCEs

Year	Composition	Ionic Conductivity	Active Materials	Electrochemical Performance	Refs.
2018	PVDF‐HFP/LiTFSI + 50 wt% LLZO + 20 µL liquid electrolyte	1.1 × 10^−4^ S cm^−1^ at 25 °C	LiFePO_4_	111 mAh g^−1^ after 180 cycles under 0.5 C at 25 °C	135
2018	PVDF‐HFP/LLZO + liquid electrolyte	3.71 × 10^−4^ S cm^−1^ at 25 °C	LiFePO_4_	153.6 mAh g^−1^ after 40 cycles under 0.2 C at 25 °C	136
2018	PVDF‐HFP + 10 wt% LAGP + EMITFSI	7.6 × 10^−4^ S cm^−1^ at 25 °C	LiFePO_4_	131 mAh g^−1^ after 50 cycles under 0.05 C at 25 °C	137
2018	A asymmetric solid electrolyte with a rigid LLZO layer (5.7 µm) modified with 7.5 nm polymer and a soft polymer layer (5.4 µm)	–	LiFePO_4_	151.2 mAh g^−1^ after 120 cycles under 0.2 C at 55 °C	113
2018	PEO/LiTFSI + 10 wt% LLZO nanowires	2.39 × 10^−4^ S cm^−1^ at 25 °C	LiFePO_4_	158.8 mAh g^−1^ after 70 cycles under 0.5 C at 60 °C	57
		1.53 × 10^−3^ S cm^−1^ at 60 °C		158.7 mAh g^−1^ after 80 cycles under 0.1 C at 45 °C	
2018	PEO/LiTFSI + LATP/PAN nanofiber network	6.5 × 10^−4^ S cm^−1^ at 60 °C	LiFePO_4_	144 mAh g^−1^ after 100 cycles under 0.2 C at 60 °C	97
2018	PEO/LiTFSI + 10 wt% LLZTO	1.17 × 10^−4^ S cm^−1^ at 30 °C	LiFePO_4_	130.2 mAh g^−1^ after 100 cycles under 0.2 C at 50 °C	44
	PEO:LLZTO:PEG:LiTFSI = 10:85:5:60	6.24 × 10^−5^ S cm^−1^ at 30 °C	LiFePO_4_	127 mAh g^−1^ after 50 cycles under 0.2 C at 50 °C	
2018	PEO/LiClO_4_ + 50 wt% LATP	9.5 × 10^−6^ S cm^−1^ at 30 °C	LiFePO_4_	109.3 mAh g^−1^ after 500 cycles under 1 C at 80 °C	104
2018	In situ PEO/LiTFSI + 2 vol% Li_3_PS_4_	8.01 × 10^−4^ S cm^−1^ at 60 °C	LiFePO_4_	116 mAh g^−1^ after 325 cycles under 0.5 C at 60 °C	138
2018	PEO + 60 wt% LLZTO + 10 wt% succinonitrile	1.22 × 10^−4^ S cm^−1^ at 30 °C	LiFePO_4_	151.1 mAh g^−1^ after 200 cycles under 0.5 C at 60 °C	139
2018	Poly(1,4‐butylene adipate)/LiClO_4_ + 70 wt% LATP	–	LiNi_0.6_Co_0.2_Mn_0.2_O_2_	A discharge capacity of 169.5 mAh g^−1^ at 55 °C	45
2018	LLZTO‐PPC‐LiTFSI	4.2 × 10^−4^ S cm^−1^ at 25 °C	LiFePO_4_/Si	A capacity retention of 82.6% after 100 cycles at room temperature and 0.1 C	121
2017	PEO/LiTFSI + 40 wt% Al‐doped LLZTO	1.12 × 10^−5^ S cm^−1^ at 25 °C	LiFePO_4_	134.9 mAh g^−1^ after 100 cycles under 0.1 C at 60 °C	50
2017	Poly(ethylene carbonate)/LiTFSI + 5 wt% LLZTO	5.2 × 10^−4^ S cm^−1^ at 20 °C	LiFePO_4_	95% after 200 cycles under 1 C at 20 °C	96
2017	PVDF‐HFP + LiTFSI + EMITFSI + LAGP	9.6 × 10^−4^ S cm^−1^ at 25 °C	LiFePO_4_	141.3 mAh g^−1^ after 50 cycles under 0.05 C at 25 °C	20
2017	PEO/LiClO_4_ + 50 wt% LLZTO	9.6 × 10^−4^ S cm^−1^ at 60 °C	LiFePO_4_	116.2 mAh g^−1^ after 500 cycles under 1 C at 60 °C	54
2017	PEO/LiTFSI + 7.5 wt% LLZO	5.5 × 10^−4^ Scm^−1^ at 25 °C	LiFePO_4_	121 mAh g^−1^ after 100 cycles under 0.5 C at 60 °C	42
2017	LLCZNO pellet and double PVDF−HFP‐based gel protected layers	–	LiFePO_4_	130.2 mAh g^−1^ after 70 cycles at a current density of 170 mA g^−1^	111
2017	LLZT‐2 wt% LiF pellet and PEO/LiTFSI‐protected layer on the anode side	–	LiFePO_4_	120 mAh g^−1^ during 100 cycles at 80 mA cm^−2^	140
			Li‐S	988 mAh g^−1^ after 100 cycles under 0.4 C at 25 °C	
2017	PEO/LiFSI + 10 vol% Al_2_O_3_ on the anode side and PEO/LiFSI + 3 vol% LICGC on the cathode side	–	Li‐S	Coulombic efficiency higher than 99% after 50 cycles under 0.1 C at 70 °C	23
2017	PVDF + 4 wt% LATP + liquid electrolyte	3.31 × 10^−4^ S cm^−1^ at 20 °C	Li‐S	458.9 mAh g^−1^ after 40 cycles under 0.4 C at 25 °C	141
2017	PEO/LiClO_4_ + 15 wt% Al^3+^/Nb^5+^ codoped LLZO	9.5 × 10^−6^ S cm^−1^ at 20 °C	Li‐S	More than 900 mAh g^−1^ at 37 °C	48
2017	PEO/LiTFSI + LAGP and PEO/LiTFSI‐modified layer on the anode side	–	LiMn_0.8_Fe_0.2_PO_4_	A high initial discharge capacity of 160.8 mAh g^−1^ and exhibits good cycling and rate performance under 0.2 C at 50 °C	99
2017	PVDF/LiClO_4_ + 10 wt% LLZTO	5 × 10^−4^ S cm^−1^ at 25 °C	LiCoO_2_	147 mAh g^−1^ after 120 cycles under 0.4 C at 25 °C	51
2017	Poly(methyl methacrylate‐styrene)/LiTFSI + LAGP	3.2 × 10^−4^ S cm^−1^ at 25 °C	Li‐O_2_	A superior long life (350 cycles, >145 days) at 50 °C	133
2016	CPMEA/LATP/CPMEA sandwich electrolyte	–	LiFePO_4_	102 mAh g^−1^ after 640 cycles under 0.6 C at 65 °C	108
2016	PEO/LiTFSI + 1 wt% Li_10_GeP_2_S_12_	1.21 × 10^−3^ S cm^−1^ at 80 °C	LiFePO_4_	137.4 mAh g^−1^ after 50 cycles under 0.5 C at 60 °C	43
2016	PEO/LiTFSI + 20 wt% LAGP	6.76 × 10^−4^ S cm^−1^ at 60 °C	LiFePO_4_	100 mAh g^−1^ after 50 cycles under 1 C at 60 °C	38
2016	PEO + 12.7 vol% LLZTO particles in size of *D*_50_ = 43 nm	2.1 × 10^−4^ S cm^−1^ at 30 °C	LiFePO_4_	345 Wh kg^−1^ (662 Wh L^−1^) under 0.1 C at 60 °C (pouch cell)	47
			LiFe_0.15_Mn_0.85_PO_4_	405 Wh kg^−1^ (700 Wh L^−1^) under 0.1 C at 60 °C (pouch cell)	

### 4.1 Typical Lithium Metal Batteries

To realize the application of SCEs in lithium metal batteries, multiple indexes of SCEs need to be evaluated, including thickness, ionic conductivity, electrochemical window, mechanical strength, potential reaction with electrode, thermal behavior, environmentally benign, and cost.^12^, ^89^ The ionic conductivity of SCEs membrane falls in the range from 10^−4^ to 10^−5^ S cm^−1^ and the electrochemical window is enhanced above 5 V versus Li^+^/Li for the incorporation of ceramic filler. A skillful design of PEO_8_‐LiTFSI‐10 wt% LLZTO (“ceramic‐in‐polymer”) and PEO‐LLZTO‐PEG‐60 wt% LiTFSI (“polymer‐in‐ceramic”) shows good flexibility, wide electrochemical window (up to 5.0 V versus Li/Li^+^), and relatively high ionic conductivity (above 10^−4^ S cm^−1^ at 55 °C).^44^ The PEO‐LLZTO composite electrolytes show strong stability with lithium anode and ability to effectively suppress lithium dendrite growth. The LiFePO_4_/Li cells using both types of composite electrolytes also present good cycle and rate performance. Besides, Li et al.^97^ applied a 3D fiber‐network‐reinforced bicontinuous composite solid electrolyte in dendrite‐free lithium metal batteries. The LiFePO_4_/Li cell with this LATP/PAN‐PEO/LiTFSI membrane presents an initial discharge capacity of 144 mAh g^−1^ with a tremendously stable coulombic efficiency of 99.5% ± 0.5% over 100 cycles at 60 °C, indicating outstanding interfacial stability between electrolyte and Li metal. The strong mechanical strength and enhanced stability on interface promote the suppression of lithium dendrite by constructing the LATP/PAN‐reinforced 3D bicontinuous structure. Furthermore, owing to the reinforced oxidation stability, the SCEs are suitable to match high‐voltage cathode materials, such as LiNi*_x_*Co*_y_*Mn_1−_ *_x_*_−_ *_y_*O_2_, LiCoO_2_, and LiMn_2_O_4_.^89^ For example, Park et al.^45^ fabricated a flexible solid electrolyte film using poly(1,4‐butylene adipate) and LiClO_4_ hybridized with Li^+^‐conductive LAGP. The as‐assembled all‐solid‐state Li/LiNi_0.6_Co_0.2_Mn_0.2_O_2_ cell using this flexible solid electrolyte film delivers an initial discharge capacity of 169.5 mAh g^−1^ with good capacity retention at 55 °C. Zhang et al.^51^ also found that the chemical structure of PVDF backbones changed greatly with presence of Li_6.75_La_3_Zr_1.75_Ta_0.25_O_12_ in PVDF/LiClO_4_ SCEs, which results in significantly improved ionic conductivity and mechanical strength. The results of experiments and first‐principle calculations indicated that the chemical dehydrofluorination of the PVDF skeleton would occur owing to the Lewis base formed by the complex between the La atom of LLZTO and N atom or C=O group of solvent molecules. The as‐prepared LiCoO_2_/Li cell with 10% LLZTO/PVDF composite membrane shows high coulombic efficiency and capacity retention of 98% after 120 cycles and high discharge capacity of 130 mAh g^−1^ at 4 C at 25 °C. The SCEs show excellent compatibility with both cathode and silicon anode.^121^ The as‐assembled Si/LLZO‐PPC/Li cells exhibit 2520, 2260, 1902, and 1342 mAh g^−1^ at 0.1 C, 0.2 C, 0.5 C, and 1 C, respectively. The Si in full Si/LFP cell shows a specific capacity of 2296 mAh g^−1^, which remains 82.6% after 100 cycles at room temperature and 0.1 C.

In all, limited by the low electrochemical stability of polymer host, most of the SCEs were applied in low‐voltage LiFPO_4_ cathode and only few were used with high‐voltage cathode such as LiNi*_x_*Co*_y_*Mn_1−_ *_x_*_−_ *_y_*O_2_. Therefore, developing novel SCEs, which could tolerate high voltage cathode, would become more valuable in the practical application of SCEs. Meanwhile, the SCEs with high ionic conductivity at room temperature will attract much more attentions in future study.

### 4.2 Li‐S Battery

Recently, Li‐S batteries have achieved great prospect for their high theoretical specific capacity (1672 mAh g^−1^), which is a magnitude higher than that of the lithiated transition‐metal oxide and phosphate cathode materials used in the state‐of‐the‐art commercial Li‐ion battery.^123^, ^124^ In addition, the sulfur is naturally abundant, easily available, and relatively cheap, all of which make it promising and significant cathode in next‐generation lithium battery.^123^, ^125^ But the dissolution and migration of intermediate lithium polysulfides in liquid electrolyte causes the loss of active materials and thus sharp capacity decay of Li‐S battery.^126^, ^127^ Seeking for the method to settle the issue of polysulfide shuttling is quite significant for the practical application of Li‐S battery. The application of the all‐solid‐state composite electrolyte seems an effective way to address the above issue.^125^, ^128^

**Figure 12:**
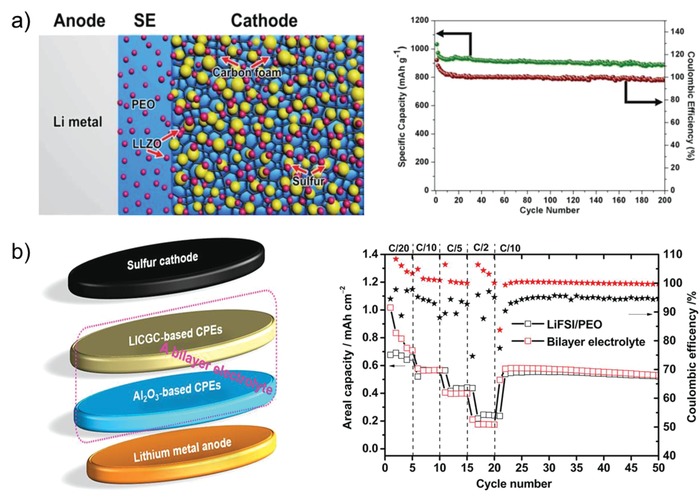
a) Schematic illustration of an all solid‐state Li‐S battery based on LLZO nanostructures. The cycling performance and coulombic efficiency of the S@LLZO@C cathode with a current density of 0.05 mA cm^−2^ at 37 °C. Reproduced with permission.^48^ Copyright 2017, American Chemical Society. b) Sketch of the Li‐S cell with a bilayer electrolyte configuration and galvanostatic cycling performance of the bilayer cell at 70 °C. Reproduced with permission.^23^ Copyright 2017, American Chemical Society.

The ceramics in SCEs could absorb lithium polysulfides via chemical bonding and thus relieve the shuttle effect. Although the shuttle effect still exists in the SCEs‐based Li‐S batteries working at high temperature caused by the high flowability of polymer at high temperature,^129^ the SCEs, as well, play an important role on improving the performance of Li‐S batteries. For example, a composite electrolyte consisting of Al^3+^/Nb^5+^ co‐doped cubic LLZO and P(EO)_8_/LiClO_4_ was prepared and applied in Li‐S battery (**Figure** **12**a). In order to increase the ion/electron conductivity of sulfur cathode, active S materials were embedded into porous carbon foam decorated by LLZO nanoparticles (S@LLZO@C). As‐prepared all‐solid‐state Li‐S battery shows acceptable specific capacity (>900 mAh g^−1^ at 37 °C) and high coulombic efficiency (approaches 100%). The LLZO nanoparticles not only act as ion‐conductive fillers but also as interfacial stabilizer to reduce the interfacial resistance.^48^ Judez et al.^23^ reported a double‐layered composite electrolyte (LICGC) consisting of NASICON‐type Li_2_O‐Al_2_O_3_‐SiO_2_‐P_2_O_5_‐TiO_2_‐GeO_2_‐LiFSI/PEO (sulfur cathode side) and Al_2_O_3_/LiFSI/PEO layers (Li metal anode side). In these all‐solid‐state Li‐S batteries, the Al_2_O_3_‐based electrolyte membrane remarkably improves the Li metal/electrolyte interface contact, resulting in high coulombic efficiency and good cycle performance (Figure 12b). Moreover, the LICGC‐based electrolyte membrane boosts the S utilization and cell areal capacity, avoiding the reaction with Li metal. The as‐fabricated Li‐S cell possesses a capacity of 518 and 0.53 mAh cm^−2^ with a coulombic efficiency higher than 99% by the end of 50 cycles at 70 °C. Therefore, the multi‐layer composite electrolytes are regarded as the promising candidates for developing all‐solid‐state Li‐S batteries with high safety and performances. The novel SCE systems, which are stable with polysulfide species and could suppress their shuttle effect effectively, are critical to promote the development of the next generation Li‐S batteries.

However, it should be noted that the specific capacity of S cathode in all‐solid‐state battery using SCEs is still quite lower and its rate and cycling performance are poor due to the larger interfacial resistance between S cathode and SCEs and severe volume expansion. Therefore, integrated S cathode/SCEs architectures with lower interfacial resistance and highly stable structure should be further developed in the future research.

### 4.3 Li‐O_2_/Air Battery

**Figure 13:**
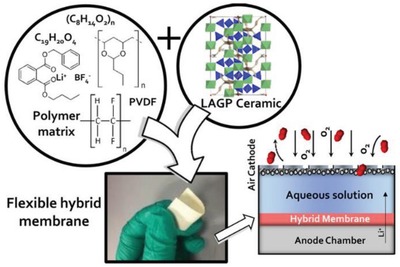
The Schematic illustration of the hybrid membrane and Li‐O_2_ cell with this membrane fabricated by Safanama et al. Reproduced with permission.^134^ Copyright 2017, American Chemical Society.

Li‐O_2_ battery is another attractive battery system for energy storage owing to its high theoretical specific energy density (≈3500 Wh kg^−1^).^130^ In spite of a great deal of reports focused on liquid and solid electrolytes used in Li‐O_2_ or Li‐air cells, few researches are investigated for the application of the SCEs these cells.^131^ The deterioration of cycling performance constantly occurred in Li‐O_2_ batteries with liquid electrolyte because of leakage and volatilization of solvents in air, which could be prospectively avoided by adopting all‐solid‐state electrolyte.^127^ Meanwhile, the solid electrolyte with high ionic conductivity is chemically stable with LiO_2_, Li_2_O_2_ and O_2_. According to previous reports, it is found that the LAGP could adsorb O_2_ owing to its open structure followed by the reduction of oxygen and the formation of LiO_2_ and Li_2_O_2_, which facilitates the process of electrochemical reaction.^132^ A solid SCE integrating the poly(methyl methacrylate‐styrene) (PMS), LAGP, and small amount of nano‐fumed SiO_2_ was also successfully fabricated and then reinforced by a polyethylene(PE) supporter.^133^ The as‐prepared PMS‐LAGP@PE shows an ion conductivity of 0.32 × 10^−3^ S· cm^−1^ at room temperature, Li^+^ transference number of 0.75 and stable electrochemical window up to 5.2 V versus Li/Li^+^. The solid‐state Li‐O_2_ battery assembled with this SCE and single‐walled carbon nanotube‐based cathode delivers long cycle life (350 cycles, >145 days) at a current density of 200 mA g^−1^ with a capacity limit of 1000 mAh g^−1^ at 50 °C. In addition, the SCE membrane is used as protective layer to protect the Li metal from reacting with liquid electrolyte and cathode material in Li‐air battery. Safanama and Adams^134^ prepared a SCE membrane consisting of polyvinyl butyral resin, PVDF, benzyl n‐butyl phthalate, LAGP, and LiBF_4_ (**Figure** **13**). This LAGP‐based SCE membrane presents high ion conductivity (1.0 × 10^−4^ S cm^−1^) and chemical stability in both acidified and neutral LiCl solutions. Due to the employment of LAGP/polymer protective layer, the aqueous/hybrid Li‐air battery cycled for 140 h with small overpotential and average energy efficiency of 93% under a current density of 0.03 mA cm^−2^.

Undoubtedly, the Li‐O_2_ or Li‐air systems using SCEs would gain remarkable performance due to the small side reactions between SCEs and cathode. Whereas, the Li‐O_2_ or Li‐air systems using SCEs present quite different reaction mechanism compared to that using liquid electrolyte. For Li‐O_2_ or Li‐air systems using liquid electrolyte, the charge/discharge reactions occur at the solid–liquid–gas interface, while which occurs at solid–gas interface in the Li‐O_2_ or Li‐air systems using SCEs. The charge/discharge reactions rate would reduce greatly due to the larger interface resistance. It is significant to develop some catalysts to promote the charge/discharge reactions and improve the reaction kinetics.

## 5 Conclusion and Perspectives

This review focuses on the research and development of the SCEs applied in all‐solid‐state lithium batteries. Although the SCEs inherit the advantages of both SPEs and ICEs, there are still some crucial drawbacks for SCEs to overcome before practical application in all‐solid‐state lithium batteries, for example, unsatisfying ionic conductivity and knotty interfacial problems. The mechanism of Li ion migration in SCEs depends on the composition and structure of SCEs. Many researches have proved that the lithium ions move fast on the surface layer between ceramics and polymer host. Therefore, the ionic conductivity of SCEs could be greatly enhanced via adjusting the size and shape of ICEs. Among all the ceramics with different morphologies such as nanowires, nanoparticles, and 3D framework, the nanowires show great improvement on the ionic conductivity of polymer electrolytes. In addition, constructing continuous and aligned express way with ceramic nanowires or 3D ceramic framework could promote the migration of lithium ions; whereas, large number of voids in 3D framework SCEs may lead to incompatible and poorly contacted interfaces, which hinders the lithium‐ion transfer to some content. At present, the ionic conductivity of SCEs could reach around 10^−4^ S cm^−1^ at room temperature, which is still much lower than that of liquid electrolytes (10^−2^ S cm^−1^) and could not well meet the requirement of practical application in all‐solid‐state lithium battery at room temperature. Thus, in order to enable the application of SCEs at room temperature, their ionic conductivity still needs to be further improved by structure design of ceramic and composition tuning of polymer matrix.

Furthermore, the interfacial issues, including interfaces between anode and SCEs, cathode and SCEs, and ceramic and polymer, are considered the crucial obstacles for the poor power and cycling performance of all‐solid‐state lithium battery. It is of great significance to constructing high stable and lower resistance for high‐performance all‐solid‐state lithium battery. The SCEs not only promote the construction of a low resistance interface between SCEs and lithium anode, but can also effectively suppress the Li dendrite growth and side reactions of ICEs with Li metal anode. Unfortunately, the thickness of the developed SCEs is still much larger than commercial separator and it is difficult to maintain the interface stability and suppress the Li dendrite growth in all‐solid‐state batteries at a higher current density and larger plating and stripping capacity. The interfacial issues at cathode side are more complex and there are numerous solid/solid interfaces with very large resistance between cathode particles and SCEs. These interfaces are unstable during long cycling of all‐solid‐state battery due to the volume change of cathode materials. Constructing high‐efficient and stable ion‐conducting networks inside the cathode is quite essential for practical application of all‐solid‐state battery. In addition, the multi‐layer symmetrical/asymmetric SCEs according to the characteristics of anode and cathode also have been developed to solve the interfacial problems of anode and cathode simultaneously. Furthermore, the homogeneous dispersion of ceramic fillers can contribute to the construction of continuous high‐speed and uniform Li ion migration channels, which may be greatly influenced by the particle size, shape, arrangement, and 3D ceramic framework of ceramic in SCEs. Some physical and chemical methods should be further developed to create compatible interface of ceramic with polymer for promoting the development and application of all‐solid‐state lithium battery.

At present, some typical lithium metal batteries, Li‐S battery, and Li‐O_2_/air battery have applied to the SCEs as electrolyte. The SCEs with prominent characteristics, including high ionic conductivity, good mechanical strength, wet interfacial contact, and satisfying thermal and electrochemical stability present preferable performances in various lithium battery systems. However, the most of the SCEs were applied in low‐voltage LiFPO_4_ cathode and only few were used with high‐voltage cathode such as LiNi*_x_*Co*_y_*Mn_1−_ *_x_*_−_ *_y_*O_2_ due to the low electrochemical stability of polymer host. It is significant to develop the high‐voltage‐resistant polymer matrix or improve the electrochemical window of polymer electrolyte using ICEs to broaden the SCE application in all‐solid‐state battery. The specific capacity of S cathode in all‐solid‐state Li‐S battery using SCEs is still quite lower and its rate and cycling performance is poor due to the larger interfacial resistance between S cathode and SCEs and severe volume expansion. Integrated S cathode/SCEs architectures with lower interfacial resistance and highly stable structure should be further developed in the future research. The charge/discharge reaction of Li‐O_2_ or Li‐air systems using SCEs would occur at solid–gas interface, which would greatly reduce the reaction kinetics due to the larger interface resistance. Some catalysts for all‐solid‐state Li‐O_2_ or Li‐air system with SCEs should be developed to promote the charge/discharge reaction kinetics and improve its rate and cycling performance.

## Conflict of Interest

The authors declare no conflict of interest.
